# Patient Satisfaction and Perceived Success with a Telephonic Health Coaching Program: The Natural Experiments for Translation in Diabetes (NEXT-D) Study, Northern California, 2011

**DOI:** 10.5888/pcd10.130116

**Published:** 2013-10-31

**Authors:** Sara R. Adams, Nancy C. Goler, Rashel S. Sanna, Mindy Boccio, David J. Bellamy, Susan D. Brown, Romain S. Neugebauer, Assiamira Ferrara, Deanne M. Wiley, Julie A. Schmittdiel

**Affiliations:** Author Affiliations: Nancy C. Goler, Rashel S. Sanna, Mindy Boccio, David J. Bellamy, Susan D. Brown, Romain S. Neugebauer, Assiamira Ferrara, Deanne M. Wiley, Julie A. Schmittdiel, Kaiser Permanente Northern California, Oakland, California.

## Abstract

**Introduction:**

Health coaching can improve lifestyle behaviors known to prevent or manage chronic conditions such as diabetes. However, little is known about the patient experience with telephonic coaching programs in real-world care settings. We examined patient satisfaction, patient’s perceived success in achieving program goals, and the patient-level correlates of these outcomes in a voluntary telephonic coaching program at a large integrated health care delivery system in northern California.

**Methods:**

Kaiser Permanente Northern California patients who participated in a telephonic coaching program in 2011 were sent a cross-sectional survey about their satisfaction with health coaching and perceived success with program goals. We examined associations with patient characteristics.

**Results:**

The survey response rate was 34%; analyses were based on the 32% who completed the survey. Of those who had completed 2 or more sessions (n = 232 [52%]), most reported being satisfied (70%) or neutral (20%) with the program, and 71% would recommend health coaching. Healthy weight, healthful eating, and physical activity were the most common topics discussed (88%). Adjusting for demographic characteristics, 73% of those who had 2 or more sessions reported that health coaching helped achieve their weight-related goal. Outcomes were positively correlated with patient activation but not consistently correlated with patient demographic characteristics.

**Conclusion:**

Levels of satisfaction and perceived success with telephonic health coaching provided by a health plan were high and positively correlated with the number of sessions completed and patient activation. Voluntary telephonic health coaching programs should promote retention and assess patients’ activation levels.

## Introduction

Health coaching is a promising strategy for helping patients make behavior modifications that can prevent or manage diabetes and other chronic conditions (1,2). Health coaching, often delivered via one-on-one telephone encounters with nonphysician providers, strengthens patient commitment to lifestyle change by using techniques such as motivational interviewing (3–8). Featuring goal-setting and enhanced coordination with health care providers, health coaching improves behaviors related to nutrition, weight management, and medication adherence (7,9,10). On the basis of this promise, health plans and providers are offering telephonic health coaching programs both to meet the demand for healthy lifestyle support and as a care-management strategy to improve outcomes and reduce costs (11,12).

In general, patients report being satisfied with medical care delivered telephonically, ie, telemedicine (13). Although several studies report high satisfaction with telephonic health coaching and specifically with coaching interventions of moderate intensity (eg, multiple sessions initiated by the coach), these programs were limited to patients with a specific condition or risk factor (9,14,15). Little is known about patient satisfaction with telephonic health coaching when implemented via low-intensity, voluntary programs providing healthy lifestyle support to a general population.

As part of the Natural Experiments for Translation in Diabetes (NEXT-D) Study testing the effectiveness of population-targeted diabetes prevention and control policies, we evaluated a telephonic health coaching program providing support for healthy weight, healthful eating, physical activity, tobacco use cessation, and stress reduction to members of a large integrated health care delivery system (16). Our aim in this preliminary analysis was to examine patient-centered outcomes of coaching, such as patient satisfaction and perceived success in achieving goals, and the patient-level correlates of these outcomes. We postulated that satisfaction and perceived success would be correlated with the coaching topic, the number of coaching sessions the patient chose to complete, and patient activation, defined as the “knowledge, skill, and confidence for self-management” (p. 1,918) (17).

## Methods

### Study setting and population

Kaiser Permanente Northern California (KPNC), an integrated health care delivery system of 48 medical facilities that provides comprehensive care to more than 3 million members, has provided a health coaching program through its centralized telephonic Wellness Coaching Center (WCC) since January 2010 (18). A total of 1,927 members participated in the first 18 months of the program. KPNC members are broadly representative of the local and statewide population (19).

### Program description

WCC coaches support patients with 5 primary lifestyle changes: more healthful eating, increased physical activity, weight management, tobacco use cessation, and stress reduction. Through collaborative conversations, wellness coaches provide encouragement, guidance, and motivation tailored to the patients’ individual interests and readiness to change. Wellness coaching is a covered benefit offered at no cost to the approximately 2 million adult KPNC members. Members are made aware of the program through referrals by KPNC providers and staff, partnerships with employers, and direct patient outreach. Participation is voluntary, although some employers have offered an incentive for participation. At the time of the survey, patients were allowed up to 4 sessions per calendar year. The program is staffed by wellness coaches who are clinical health educators trained in motivational interviewing. Motivational interviewing, a patient-centered counseling style for addressing the common problem of ambivalence about change (20), has been effective for both weight loss and substance abuse (21,22). At the time of this study, all 4 wellness coaches were English-speaking and 1 was Spanish-speaking.

Patients participate by first scheduling an appointment with a wellness coach, and coaches call patients at their designated time. A typical coaching engagement consists of 1 initial session (20–30 minutes) and shorter follow-up contacts (10–20 minutes). Wellness coaches document the sessions in the member’s electronic medical record (EMR) with details about the referral source (eg, physician, employer), whether the member has completed items on a preventive health checklist (eg, recommended screenings, vaccinations), the topic(s) that the member discussed, the member’s readiness to change health behaviors (measured on a self-report scale from 0 to 10, with 0 being “not ready” and 10 being “very ready”), and the member’s progress in reaching the health goal starting at the second session (ie, plan attempted, completed, or not attempted). Wellness coaches are able to directly coordinate care with members’ KPNC physicians (eg, requesting a prescription for smoking cessation medications) and refer members to other supportive KPNC services (eg, health education classes).

### Survey methods

We conducted a cross-sectional, observational survey of patients who had their first wellness coaching session between January 1, 2011, and August 23, 2011. Patients were excluded from the survey if their primary language was not English, the patient had recently died, the patient had previously requested not to be contacted for research activities, or the patient’s primary address was not in California. We administered the survey with an adapted Dillman method (23): an initial survey and cover letter were mailed in October 2011, followed 1 week later by a reminder letter and 3 weeks later by a reminder cover letter and a second copy of the survey. Patients had the choice to complete the 4-page survey on paper or online.

We present the frequency of outcomes stratified by the individual topic discussed (healthy weight, healthful eating, physical activity, tobacco use cessation, and stress reduction) and by a summary variable indicating that any of the weight-related topics were discussed (namely, healthy weight, healthful eating, or physical activity). Patients were determined to have discussed a topic if they marked that topic on the survey or the wellness coach had indicated that topic was discussed at any session. The survey contained 2 outcome domains: patient satisfaction (ie, overall satisfaction and willingness to recommend the program) and perceived success (ie, patient report of whether wellness coaching helped with goal achievement). The first outcome was a 3-level indicator for satisfaction (respondent indicated being satisfied/extremely satisfied vs neutral vs dissatisfied/extremely dissatisfied to the question, “Overall, how satisfied are you with the service you received from the Wellness Coach?”). The second outcome was a 3-level indicator for willingness to recommend the program (agree/strongly agree vs neither agree or disagree vs disagree/strongly disagree with the statement, “I would recommend Wellness Coaching to a family member, friend or colleague”). The third outcome was perceived success of health coaching for those who called to discuss a weight-related topic, coded as a dichotomous variable (1 for yes to the question “Did Wellness Coaching help?” for any of the 3 weight-related topics, 0 if the respondent indicated no, don’t know, or left the question blank). In addition, patients were asked if their reasons for participating in wellness coaching included to “improve your health,” “improve your quality of life,” “reduce your risk of disease,” or “address a recent health concern” and whether wellness coaching helped with these goals.

The survey solicited information about patient demographics (race/ethnicity, education, and income) and patient activation as measured by the Patient Activation Measure 6 (PAM-6) (24), a version of the well-validated Patient Activation Measure 13 (17) that was modified by the developers. Patient activation has been positively correlated with healthy behaviors, better health outcomes, and better health care experiences (25). We expected the level of patient activation to be positively correlated with program satisfaction and perceived success (26). Raw PAM-6 scores were mapped to 4 patient activations levels: “May not yet believe that the patient role is important” (level 1); “Lacks confidence and knowledge to take action” (level 2); “Beginning to take action” (level 3); and “Has difficulty maintaining behaviors over time” (level 4) (24).

Additional patient characteristics for respondents and nonrespondents, including age at the first coaching session, sex, race/ethnicity, and body mass index (BMI; calculated as the weight in kilograms divided by the height in meters squared) at the most recent visit before the first coaching session, were obtained from the EMR. Because the EMR does not contain individual-level socioeconomic variables, we created categorical variables representing the median annual household income (<$30,000; $30,000–$79,999; $80,000–$119,999, or ≥$120,000) and average educational attainment (<15%, 15%–29%, 30%–44%, or ≥45% with a bachelor’s degree) for each patient’s US Census 2000 block group of residence. The dates and topics of the wellness coaching sessions were assessed from the EMR at the time of the first survey mailing.

The patient activation measure is associated with key process and health outcomes measures (27). On the basis of this, we conservatively calculated that a sample size of 436 would be sufficient to determine a 5-point difference in mean PAM-6 scores between women and men or between respondents discussing weight-related topics and those discussing other topics, using an estimate that the ratio of women to men and the ratio of weight-related topics discussed to other topics would both be 3:1. The sample size calculation was based on an α level of .05 (2-tailed), 80% power, and PAM-6 standard deviation of 16.1.

### Statistical analyses

Two sample *t* tests, *F* tests, and Pearson χ^2^ tests of independence were used to compare the characteristics of respondents with those of nonrespondents. We used Pearson χ^2^ tests of independence to assess bivariate associations between survey outcome measures and the categorical indicators: sex, race/ethnicity (non-Hispanic white vs other/missing), number of sessions (1 vs ≥2), and topic of first wellness coaching session (weight-related topic vs other/missing). We calculated mean age, BMI, and PAM-6 activation scores per stratified outcome level (eg, the mean age for patients who reported being satisfied was compared with the mean age for patients who reported being dissatisfied), and evaluated potential associations with *t* tests and *F* tests. All tests were 2-sided with an α level of .05 to determine significance.

The predicted percentages of patients reporting satisfaction and perceived success with wellness coaching were generated from logistic regression models in which patient characteristics (age, sex, race/ethnicity, BMI, education, income, PAM-6 activation level, and number of sessions) were set to their mean or proportional values. We also calculated predicted percentages for all patients who discussed a weight-related topic and stratified by the patient characteristics that consistently correlated with the outcome variables in the bivariate analyses. We used SAS software versions 9.1.3 and 9.3 (SAS Institute Inc, Cary, North Carolina) for all data management and analysis. The KPNC Institutional Review Board approved this study.

## Results

Of the 1,539 patients who had their first wellness coaching session during the study period, 112 were excluded from the eligible survey cohort because they had a primary language other than English (n = 101), had previously requested to be excluded from research (n = 6), had recently died (n = 2), or had a primary mailing address not in California (n = 3). The remaining patients (n = 1,427) met the inclusion criteria and were sent the initial mailing. Of these, 17 people were later determined to be ineligible due to undelivered survey (n = 15), recent death (n = 1), or not being a KPNC member (n = 1). A total of 486 patients returned a survey; the resulting response rate was 34% (486/1,410). Respondents and nonrespondents were similar with regard to sex, geocoded education, and topic of first wellness coaching session. Compared with nonrespondents, respondents to the survey were more likely to be older, to be white, to have lower BMI, and to have attended more than 1 WCC session (Table 1). Participation in wellness coaching had been identified through the EMR, yet when asked “Have you had any phone calls with a Kaiser Permanente Wellness Coach?” 40 respondents indicated no, don’t know, or “I choose not to answer this question” and did not complete the rest of the survey. The main analyses were based on the remaining 446 respondents.

**Table 1:** Patient Characteristics for the Respondents and Nonrespondents to the Kaiser Permanente Wellness Coaching Program Survey, Northern California, 2011

Characteristic	Respondents (n = 446)	Nonrespondents (n = 924)	*P* Value^a^
**Female, n (%)**	372 (83)	741 (80)	.15
**Age, y, mean (SD)**	56 (14)	48 (14)	<.001
18–39, n (%)	59 (13)	261 (28)	<.001
40–49, n (%)	80 (18)	217 (23)	
50–59, n (%)	116 (26)	250 (27)	
60–69, n (%)	121 (27)	142 (15)	
≥70, n (%)	70 (16)	54 (6)	
**Race/ethnicity, n (%)**			.003
White/Caucasian, non-Hispanic	236 (53)	392 (42)	
Black/African American, non-Hispanic	57 (13)	151 (16)	
Spanish/Hispanic/Latino	48 (11)	113 (12)	
Asian/Native Hawaiian/Pacific Islander, non-Hispanic	26 (6)	72 (8)	
Other or multiracial, non-Hispanic	24 (5)	37 (4)	
Unknown/choose not to answer	55 (12)	159 (17)	
**Body mass index,^b^ mean (SD)**	32.4 (8.0)	33.7 (8.8)	.007
<25 (normal), n (%)	68 (15)	108 (12)	.02
25–29 (overweight), n (%)	111 (25)	217 (23)	
≥30 (obese), n (%)	253 (57)	538 (58)	
Missing, n (%)	14 (3)	61 (7)	
**Total no. of wellness coaching sessions, mean (SD)**	1.8 (1.0)	1.5 (0.8)	<.001
1, n (%)	214 (48)	644 (70)	<.001
2, n (%)	134 (30)	182 (20)	
3, n (%)	54 (12)	59 (6)	
≥4, n (%)	44 (10)	39 (4)	
**Topic of first session, n (%)**			.07
Healthy weight	235 (53)	479 (52)	
Healthful eating	77 (17)	132 (14)	
Physical activity	53 (12)	87 (9)	
Reduce stress	43 (10)	104 (11)	
Quit tobacco use	28 (6)	94 (10)	
Other/missing topic	10 (2)	28 (3)	
**Topic of first session was weight-related (any of weight/eating/activity), n (%)**	365 (82)	698 (76)	.009
**Education, n (%)**			
High school graduate/GED or less	34 (8)	NA	
Technical school, some college, or 2-year degree	149 (33)		
College graduate	118 (27)		
Graduate school	106 (24)		
Other/missing/choose not to answer	39 (9)		
**Annual income, $, n (%)**			
<30,000	69 (15)	NA	
30,000–79,999	148 (33)		
80,000–119,000	74 (17)		
≥120,000	50 (11)		
Missing/choose not to answer	105 (24)		
**PAM-6 activation level, mean score (SD)**	62.8 (17.4)	NA	
Level 1: may not yet believe that the patient role is important, n (%)	83 (19)	NA	
Level 2: lacks confidence and knowledge to take action, n (%)	111 (25)		
Level 3: beginning to take action, n (%)	100 (22)		
Level 4: has difficulty maintaining behaviors over time, n (%)	139 (31)		
Level missing	13 (3)		

Abbreviation: SD, standard deviation; NA, not applicable; GED: general educational development certificate; PAM, Patient Activation Measure 6 (24).

a Determined by a χ^2^ test for the difference in proportions or a *t* test for the difference in means.

b BMI is calculated as weight in kilograms divided by height in meters squared.

Of the people who had participated in wellness coaching and completed the survey, most were women (83%), of white race/ethnicity (53%), and overweight (25%) or obese (57%) (Table 1). Most discussed a weight-related topic at their first coaching session (82%). A total of 232 patients (52% of the analysis cohort; 16% of all patients who were mailed a survey) had completed more than 1 coaching session at the time of the survey.

In the overall sample, most respondents indicated that they were satisfied or extremely satisfied (60%) or neutral (24%) with the service received from the wellness coach (Table 2), and 64% would recommend wellness coaching to others. Most respondents were more aware of Kaiser Permanente resources (57%) and more likely to participate in other Kaiser Permanente programs (54%) after speaking with a wellness coach. Most patients reported that wellness coaching helped them achieve their goal (50%–62%, depending on the topic). Respondents with 2 or more sessions were more likely than respondents with 1 session to be satisfied (*P* < .001) and to recommend wellness coaching (*P* = .007). Respondents who completed 2 or more sessions were more likely to report that wellness coaching helped them to eat more healthfully (68% vs 54%, *P* = .04), increase their physical activity (71% vs 45%, *P* < .001), improve their health (79% vs 61%, *P* = .005), improve their quality of life (83% vs 61%, *P* < .001), and reduce their risk of disease (73% vs 51%, *P* = .01).

**Table 2:** Survey Results for Respondents (n = 446) to the Kaiser Permanente Wellness Coaching Program Survey, Overall and Stratified by the Number of Sessions Completed, Northern California, 2011

Characteristic	Overall, N (%)	1 Session, N (%)	≥2 Sessions, N (%)	*P* Value^a^
**Satisfaction outcomes**				
**Patient’s overall satisfaction with the service received from the wellness coach^b^**				
Satisfied/extremely satisfied	264 (60)	104 (50)	160 (70)	<.001
Neutral	104 (24)	59 (28)	45 (20)	
Dissatisfied/extremely dissatisfied	70 (16)	45 (22)	25 (11)	
**Patient would recommend wellness coaching to a family member, friend, or colleague^b^**				
Agree/strongly agree	273 (64)	114 (56)	159 (71)	.007
Neither agree or disagree	83 (19)	45 (22)	38 (17)	
Disagree/strongly disagree	71 (17)	43 (21)	28 (12)	
**Patient reported the following was adequate:**				
Number of sessions offered	225 (50)	92 (43)	133 (57)	.002
Amount of time spent with the coach	319 (72)	140 (65)	179 (77)	.006
**Perceived success outcomes**				
**Topic discussed with a wellness coach**				
Healthy weight	321 (72)	145 (68)	176 (76)	.06
Healthful eating	203 (46)	92 (43)	111 (48)	.30
Physical activity	181 (41)	77 (36)	104 (45)	.06
Weight-related (any of 3 listed above)	392 (88)	185 (86)	207 (89)	.37
Reduce stress	100 (22)	43 (20)	57 (25)	.26
Quit tobacco use	34 (8)	13 (6)	21 (9)	.24
**Patient reported wellness coaching helped for the following topics (among those who discussed the topic)**				
Achieve or maintain a healthy weight	141 (44)	60 (41)	81 (46)	.40
Eat more healthfully	126 (62)	50 (54)	76 (68)	.04
Increase physical activity	109 (60)	35 (45)	74 (71)	<.001
Weight-related (any of 3 above)	232 (59)	95 (51)	137 (66)	.003
Reduce stress	50 (50)	17 (40)	33 (58)	.07
Quit tobacco use	18 (53)	7 (54)	11 (52)	.93
**Patient reported wellness coaching helped (among those who gave that reason for participating)**				
Improve health	158 (71)	59 (61)	99 (79)	.005
Improve quality of life	131 (73)	48 (61)	83 (83)	<.001
Reduce risk of disease	81 (62)	33 (51)	48 (73)	.01
Address a recent health concern	77 (62)	28 (53)	49 (69)	.07
**Other outcomes**				
**Patient was more aware of Kaiser Permanente resources after wellness coaching^a^**				
Agree/strongly agree	248 (57)	111 (53)	137 (61)	.02
Neither agree or disagree	101 (23)	45 (22)	56 (25)	
Disagree/strongly disagree	86 (20)	53 (25)	33 (15)	
**Patient was more likely to participate in other Kaiser Permanente programs after wellness coaching^b^**				
Agree/strongly agree	235 (54)	99 (48)	136 (60)	.004
Neither agree or disagree	134 (31)	67 (32)	67 (30)	
Disagree/strongly disagree	63 (15)	41 (20)	22 (10)	

a Determined by a χ^2^ test for the difference in proportions between patients with 1 session and those with 2 or more sessions.

b Denominators for percentages are total responses in the category, not total survey respondents.

PAM-6 scores significantly increased with increasing levels of satisfaction and likelihood of recommending the WCC program (Table 3). The patients who reported that wellness coaching helped them achieve their weight-related goal also had significantly higher PAM-6 scores compared with those who did not report that wellness coaching helped.

**Table 3:** Mean Patient Activation Measure 6 Score by Satisfaction and Perceived Success Response Level Among Respondents (n = 446) to the Kaiser Permanente Wellness Coaching Program Survey, Northern California, 2011

Characteristic	PAM-6 Score, Mean (SD)	*P* Value^a^
**Satisfaction outcomes**		
**Patient’s overall satisfaction with the service received from the wellness coach**		
Satisfied/extremely satisfied	66.9 (17.5)	<.001
Neutral	57.8 (15.7)	
Dissatisfied/extremely dissatisfied	55.6 (15.0)	
**Patient would recommend wellness coaching to a family member, friend, or colleague**		
Agree/strongly agree	65.8 (17.6)	<.001
Neither agree or disagree	58.9 (14.1)	
Disagree/strongly disagree	59.1 (18.4)	
**Patient reported the number of sessions offered was adequate**		
Yes	67.4 (17.8)	<.001
No	58.2 (15.8)	
**Patient reported the amount of time spent with the coach was adequate**		
Yes	65.3 (17.3)	<.001
No	56.5 (16.0)	
**Perceived success outcome**		
**Patient reported wellness coaching helped for a weight-related goal (among those who discussed a weight-related topic)**		
Yes	66.3 (17.4)	<.001
No	56.2 (15.3)	
**Other outcome**		
**Patient was more aware of Kaiser Permanente resources after wellness coaching**		
Agree/strongly agree	65.3 (17.3)	.003
Neither agree or disagree	61.6 (16.0)	
Disagree/strongly disagree	58.2 (18.1)	

Abbreviation: PAM, Patient Activation Measure 6 (24); SD, standard deviation.

a Determined by either a *t* or *F* test for the difference in mean PAM-6 scores by patient response.

Satisfaction and perceived success were not consistently correlated with the other indicators tested (ie, sex, race/ethnicity, topic of first session, age, and BMI). The few exceptions were that satisfied respondents tended to be older than those who were not satisfied (mean [standard deviation] 58 [14] years vs 54 [14] years, *P* = .01), and white respondents were more likely than nonwhite respondents to recommend wellness coaching to others (71% vs 55%, *P* = .002). Men were also more likely than women to report being more aware of Kaiser Permanente resources after wellness coaching (74% vs 54%, *P* = .005).

**Figure 1:**
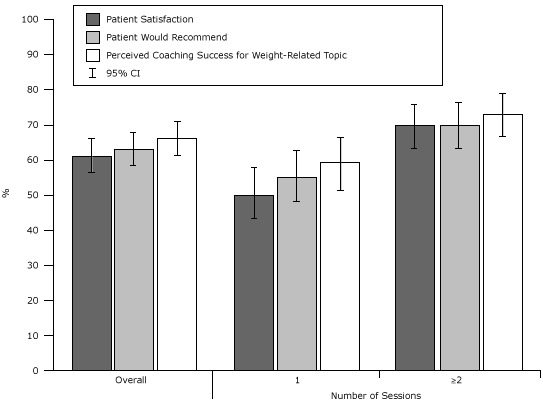
Predicted percentage of patient satisfaction and perceived success for a weight-related topic, stratified by the number of coaching sessions completed, Kaiser Permanente Wellness Coaching Program Survey, northern California, 2011. Percentages are adjusted for age, sex, race/ethnicity, body mass index, education, and income. Abbreviation: CI, confidence interval. **Table:** 

**Figure 2:**
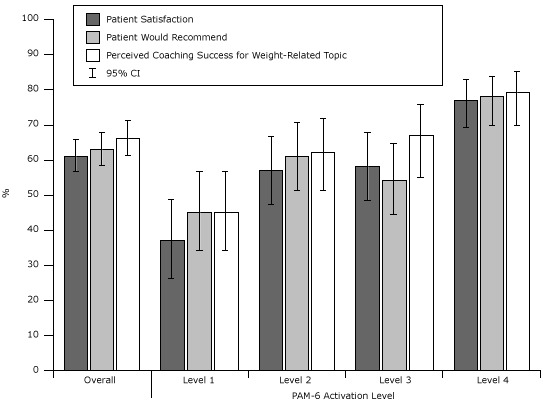
Predicted percentage of patient satisfaction and perceived success for a weight-related topic, stratified by Patient Activation Measure 6 (PAM-6) (24) activation level, Kaiser Permanente Wellness Coaching Program Survey, northern California, 2011. Percentages are adjusted for age, sex, race/ethnicity, body mass index, education, and income. Abbreviation: CI, confidence interval. **Table:** 

Predicted percentages of the primary outcomes were calculated for the overall cohort as well as stratified by the number of sessions completed (1 vs ≥2; Figure 1) and PAM-6 activation level (levels 1–4; Figure 2). After controlling for the number of sessions, age, sex, race/ethnicity, BMI, education, and income, patients at the highest activation level were significantly more likely than those at the lowest activation level to report that they were satisfied with the program (77% vs 37%, *P* < .001) and that wellness coaching helped them address a weight-related goal (79% vs 45%, *P* < .001).

No. of Sessions	Patient Satisfaction, % (95% CI)	Patient Would Recommend, % (95% CI)	Perceived Coaching Success for Weight-Related Topic, % (95% CI)
Overall	61 (56–66)	63 (58–68)	66 (61–71)
1	50 (43–58)	55 (48–63)	59 (51–66)
≥2	70 (63–76)	70 (63–76)	73 (66–79)

PAM-6 Activation Level	Patient Satisfaction, % (95% CI)	Patient Would Recommend, % (95% CI)	Perceived Coaching Success for Weight-Related Topic, % (95% CI)
Overall	61 (56–66)	63 (58–68)	66 (61–71)
1	37 (26–49)	45 (34–57)	45 (34–57)
2	57 (47–67)	61 (51–71)	62 (51–72)
3	58 (48–68)	54 (44–65)	67 (55–76)
4	77 (69–83)	78 (70–84)	79 (70–85)

## Discussion

Our survey of Kaiser Permanente patients participating in a health coaching program indicated most were satisfied with the program and believed that coaching had helped them achieve their health-related goals. These patient-reported satisfaction and success measures were positively correlated with the number of coaching sessions completed but not consistently correlated with age, sex, race/ethnicity, BMI, education, income, or the topics discussed. Previously published studies have only looked at satisfaction with health coaching when it was delivered as part of a multifaceted wellness program (28) or focused on a small group with a specific disease that received more intensive coaching (9,14,15,29). Our study is the first to assess satisfaction with voluntary health coaching offered to all adult members of a health plan regardless of pre-existing comorbidities. Our results suggest that population-based telephonic coaching programs can be an effective approach to helping patients achieve their wellness goals.

Our study found that patients attending 2 or more coaching sessions were more satisfied with the coaching program and reported higher levels of self-perceived success. Many patients in this study attended only 1 coaching session out of the 4 that they were eligible to attend. It is unclear if more motivated patients signed up for further visits or if that by attending more visits, they were further motivated to change. Although these results are cross-sectional and cannot determine causality, our study suggests voluntary coaching programs should focus on retention to improve success. This relationship would be important to discern in future studies.

After controlling for demographic variables, patients at the highest activation level were significantly more likely than those at the lowest activation level to report that they were satisfied with the program and that wellness coaching helped them address a weight-related goal. Because patient activation was not measured at baseline, we cannot determine whether patient’s activation levels were influenced by wellness coaching participation. Coaching programs may benefit from measuring patient activation before and following coaching participation to tailor the coaching interaction to the patient’s activation level and to study whether wellness coaching modifies activation levels.

Several limitations should be noted. The survey response rate was low (34%), and respondents differed from nonrespondents in age, race/ethnicity, BMI, and number of sessions attended. Although outcomes were not correlated with age, race/ethnicity, or BMI, the results may be biased because nonrespondents were more likely than respondents to have attended only 1 WCC session, and attending more coaching sessions was associated with higher satisfaction and perceived success. Participants in the WCC program were predominantly women and approximately half were white, and this limits the generalizability of our findings to all patients who participate in coaching programs. A small number of participants may have received an incentive from their employer to enroll in the voluntary WCC program. In addition, our survey was cross-sectional and therefore we cannot determine causality. Some of the patients were surveyed more than 6 months after their initial session, so their impression of wellness coaching might have been influenced by other factors in the time following their interaction with a wellness coach. Satisfaction with health care is an important patient-centered outcome (30), but objective clinical outcomes should also be examined. Further studies of WCC participants will evaluate success based on clinical measures available in the EMR (ie, BMI and systolic blood pressure).

Health coaching is a population-based approach to encourage healthy lifestyle choices and prevent chronic disease that has been embraced by health care delivery systems. Our study found that levels of satisfaction and perceived success with health coaching provided by a health plan were high and positively correlated with the number of sessions completed and patient activation. Health coaching appears promising but needs to be studied further to maximize health promotion outcomes.
